# Galactomannan and Zymosan Block the Epinephrine-Induced Particle Transport in Tracheal Epithelium

**DOI:** 10.1371/journal.pone.0143163

**Published:** 2015-11-16

**Authors:** Sebastian Weiterer, Thomas Kohlen, Florian Veit, Lydia Sachs, Florian Uhle, Christoph Lichtenstern, Markus A. Weigand, Michael Henrich

**Affiliations:** 1 Department of Anaesthesiology, Heidelberg University Hospital, Heidelberg, Germany; 2 Department of Anaesthesiology and Intensive Care Medicine, Justus-Liebig-University Giessen, Giessen, Germany; 3 Excellencecluster Cardio-Pulmonary System (ECCPS), German Lung Center (DZL), Universities of Giessen and Marburg Lung Center (UGMLC), Justus-Liebig-University Giessen, Giessen, Germany; 4 Department of Anaesthesiology and Intensive Care Medicine, St. Vincentius-Clinic, Karlsruhe, Germany; University of North Dakota, UNITED STATES

## Abstract

**Background:**

Ciliary beating by respiratory epithelial cells continuously purges pathogens from the lower airways. Here we investigated the effect of the fungal cell wall polysaccharides Galactomannan (GM) and Zymosan (Zym) on the adrenergic activated particle transport velocity (PTV) of tracheal epithelium.

**Methods:**

Experiments were performed using tracheae isolated from male C57BL/6J mice. Transport velocity of the cilia bearing epithelial cells was measured by analysing recorded image sequences. Generation of reactive oxygen species (ROS) were determined using Amplex Red reagents. PCR experiments were performed on isolated tracheal epithelium to identify adrenergic receptor mRNA.

**Results:**

The adrenergic receptors α1D, α2A, β1 and β2 have been identified in isolated tracheal epithelium. We found epinephrine responsible for an increase in PTV, which could only be reduced by selective β-receptor-inhibition. In addition, either GM or Zym prevented the epinephrine induced PTV increase. Furthermore, we observed a strong ROS generation evoked by GM or Zym. However, epinephrine induced increase in PTV recovered in the presence of GM and Zym after application of ROS scavengers.

**Conclusion:**

Both GM or Zym trigger reversible ROS generation in tracheal tissue leading to inhibition of the β-adrenergic increase in PTV.

## Introduction

The mucociliary apparatus of the lower respiratory tract plays a pivotal role in clearing and protection of airway surface areas from debris, infectious particles and pathogens. Ciliary bearing cells are located in the airway epithelium to transport impurities containing mucus in oral direction of the bronchoalveolary system [1]. However, different factors can affect this important mechanical defense mechanism. Dysfunction of fluid and electrolyte transport caused by loss of cystic fibrosis transmembrane conductance regulator (CFTR) results in impaired mucus clearance and susceptibility to airway infection [2–7].

As much as the quality of mucus, the transport efficacy of the ciliated cells seems to play an important role in the mucociliary system. These cells are under continuous control of several regulating factors, for example cyclic adenosine monophosphate (cAMP), cyclic guanosine monophosphate (cGMP) as well as high intracellular calcium concentrations [8]. Further the physiological ciliary activity is constantly adjusted by neuromodulators like acetylcholine, noradrenaline or serotonin [9]. Adrenergic regulation plays a key role in ciliary beat activation. Catecholamines supplied from the circulation or from autonomic nerve endings in the tracheal wall can increase ciliary beat frequency. Because β1- and β2-adrenergic receptors are expressed in both the tracheal epithelium and its underlying muscle layers [10] [11], catecholamines can stimulate ciliary beating from either mucosal or serosal direction [12]. Thus, adrenergic stimulation functions in host defense by increasing basal ciliary activity to prevent contamination of deeper airways and alveoli. Infectious and inflammatory stimuli also could influence ciliary function. The cytokine tumor necrosis factor-α (TNF-α), which is generated during inflammation, enhances the mucociliary transport via serotonin that is released from epithelial mast cells [13]. This mechanism is understood as an early patho-physiological response. There is emerging data that pathogenic fungi not only activate the immune system in lungs by triggering the generation of cytokines but also promote bacterial infections of lower airways [14] [15]. Since ciliated cells belong in a broader sense to the immune system, it seems possible that fungi have evolved mechanisms to impair ciliary transport. Impaired ciliary function by fungi could subsequently enable bacterial infection. Fungal colonization of airways can be diagnosed by cell wall components in broncho-alveolar-fluid samples [16]. These components, which are released from fungal cells, may directly alter the mucociliary clearance mechanism. Here we investigated the effect of Galactomannan (GM), a polysaccharide derived from Aspergillus fumigatus cell wall, and Zymosan (Zym) a cell-wall extract of Saccharomyces cerevisiae [17] on ciliary clearing mechanism. Both GM and Zym stimulate innate immune responses [18], however their effects onto the function of adrenaline activated tracheal ciliary epithelial cells is still unclear.

## Materials and Methods

### Preparation of mice trachea

The experimental protocol was approved by the Animal Welfare Office of the Justus-Liebig-University Giessen (Permit Number: 570_AZ).

Male C57BL6J (Charles River) mice (12–15 weeks old; 25–35 g) were euthanized by an overdose of isoflurane (Baxter, Unterschleißheim, Germany) in a closed chamber. After confirmation of death, a parasternal incision was made followed by removal of the submandibular gland and the infrahyoidal musculature. Tracheae were explanted immediately after opening the thorax and dissected out by gently cutting the tracheae directly cranial of the bifurcation and caudal of the larynx. The tracheae were then immediately mounted with two minutiae (Fiebig Lehrmittel, Berlin, Germany) in a Delta T culture dish (Bioptechs, Butler, PA, USA) bottom coated with Sylgard polymer (Dow Corning, Wiesbaden, Germany) filled with HEPES-buffer (2 ml; 4°C; pH 7.4) covering the whole trachea. The musculus trachealis was facing upwards and longitudinally cut after fine preparation.

### RNA extraction

For RNA isolation the tracheae were homogenized in a bead mill treaded with TRIzol (LifeTechnologies, Carlsbad, USA). RNA extraction was conducted using guanidinium thiocyanate-phenol-chloroform separation as described before [13]. Two different column-based RNA extraction kits were used for the following processing. RNeasy Plus Mini Kit was used for RNA extraction from whole tracheae, RNeasy Plus Micro Kit for RNA extraction from the isolated epithelial cells (both from Qiagen, Hilden, Germany). For RNA analysis of the tracheal epithelium we gently scrubbed the epithelial layer from isolated and opened tracheae using a hygienic swab (Raucotupf, Lohmann & Rauscher GmbH & Co. KG, Neuwied, Germany). The swab tip was homogenized in a bead mill with 350 μl of RLT Plus Buffer (Quiagen, Hilden, Germany) and 3.5 μl 2-Mercaptoethanol (Carl Roth, Karlsruhe, Germany).

### RT-PCR

RNA was quantified using the Nanodrop system (Thermo Scientific, Waltham, USA) and 0.25 μg of total RNA was used in the reaction. The synthesis step was adjusted to 20 min in order to ensure proper cDNA synthesis even in the presence of secondary structures. Reactions without enzyme where used as negative controls (“-RT”). The obtained cDNA was stored at –80°C until further PCR experiments.

For subsequent PCR analysis, the GoTaq Flexi Kit (Promega, Madison, USA) was used. Primer concentration was adjusted to 0.2 μM each in the final reaction mix, MgCl_2_ concentration and number of cycles was optimized for each primer pair separately (supporting information S1 Table). PCR reactions were performed in a Mastercycler gradient (Eppendorf, Hamburg, Germany) with the following protocol: After initial polymerase activation at 95°C for 2 min, the denaturing step was performed at 95°C for 1 min, followed by a 1 min annealing step at primer pair-specific temperature and a final extension step at 56°C for 1 min. For each target gene, positive control reactions using RNA isolated from mesenteric vessels (α1), brain (α2), heart (β1, β2) and fat (β3) were used.

PCR products were analyzed by agarose gel electrophoresis (2.5% TAE). 25 μl of each PCR reaction mix was loaded per lane and separation was performed applying 100 V for 60 min. Visualization of PCR products was achieved after staining of the gel with Sybr Safe (Life Technologies, Carlsbad, USA) for 40 min with a digital imaging system (Vilber Lourmat, Eberhardzell, Germany). GeneRuler 100 bp DNA Ladder (Thermo Scientific, Waltham, USA) was conducted for sizing and quantification of DNA.

### Histological staining

For histological staining, isolated organs were transferred into 4% paraformaldehyde (PFA) in 0.1 M phosphate buffer solution at pH 7.2 and stored overnight for immersion fixation at 4°C. After fixation the tissue was rinsed repeatedly in 0.1 M phosphate buffer and cryoprotected overnight in the same buffer supplemented with 18% sucrose. The tracheal specimens were snap frozen on filter paper in upright position in Tissue-Tek (Sacura, Finetek, Torrance, USA) using liquid nitrogen. Transverse cryosections of 10 μm thickness were processed using a cryostat (CM1900; Leica, Wetzlar, Germany). Sections were mounted on glass slides and coverslipped air dried. Afterwards the sections were briefly stained with filtered Richardson solution (0.5 g Azur II, 0.25 g methylene blue, 0.25 g Borax in 100 ml distilled water), and then rinsed repeatedly in 0.1 M phosphate buffer and finally the sections were coverslipped in carbonate-buffered glycerol (pH 8.6). The sections were evaluated with an upright light microscope (Axioplan 2 imaging; Zeiss, Oberkochen, Germany).

### Measurement of ROS generation

H_2_O_2_ released from the trachea was detected using the Amplex Red Hydrogen Peroxide/Peroxidase Assay Kit (Life Technologies, Carlsbad, USA). The trachea was prepared as described above and equilibrated in fresh HEPES buffer (2 ml; 30°C; pH 7.4) for 60 min. After equilibration Zym or GM was added. Sampling was done directly after administration of Zym (0.1 mg/ml) or GM (0.02 mg/ml) (60 min), after 75 min (15 min after administration), and after 90 min (30 min after administration). Samples were incubated with 10 μM Amplex^®^ Red reagent (10-acetyl-3,7-dihydroxyphenoxazine) and 20 mM H_2_O_2_ working solution to detect H_2_O_2_ release from tissue. Fluorescence was measured using a microplate reader equipped for excitation in the range of 530–560 nm and fluorescence emission detection at ~590 nm (Infinite^®^ M200, Tecan).

### Imaging and measurement of particle transport velocity

After fine preparation the dish with the mounted trachea was placed on the stage of a temperature controlled upright transmission light microscope (BX50 WI; Olympus, Hamburg, Germany). The 4°C HEPES buffer was replaced by 2 ml of fresh HEPES buffer pH 7.4 (30°C). The temperature in the center of the dish was constantly regulated and controlled at 30°C. PTV was estimated using Till Vision imaging software (Till Photonics, Gräfeling, Germany) and Image Pro Plus analysis software (Media Cybernetics, Warrendale, USA) as described before [13], [19]. 3 μl of small particles, Dynabeads with 2.8–4.5 μm in diameter (Dynal Biotech GmbH, Hamburg, Germany), were applied prior to each experiment. An area of interest between 2 cartilages on the inner side of the ventral trachea was chosen using a 20x water immersion lens (BX50 WI; Olympus, Hamburg, Germany) in bright field mode focusing the particles right above the tracheal surface (position was maintained for entire experiment).

For each time point a minimum of at least 150 particles were automatically tracked. The particle transport was computerized determined by recording 200 images during a period of 16.726 s at pre-defined time points (1 image/ 83.63 ms). A background subtraction was performed pixel-by-pixel excluding non-moving objects. The particle transport velocity per time point was calculated as a mean velocity of the tracked particles.

The general organ vitality and beating potential of the ciliary cells was assured by application of ATP (10^−5^ M) at the end of each experiment, which induces an almost maximum increase in PTV (data not shown).Preparation and microscope transfer was limited within 30 min followed by a resting period (30 min) to assure baseline conditions. For each experiment the time point after the resting period was defined as minute 0, the PTV at this point was arbitrarily set as 100% to compensate the individual variability.

### Drugs and buffer solutions

ATP, Clonidine, GM, N-Acetyl-L-cysteine (ACC), Tempol all from Sigma (Deisenhofen, Germany), Zym (InvivoGen, San Diego, USA), Milrione (Hikma, Portugal). 2-Deoxyadenosine monohydrate (2-DAM), CPG 20712, ICI 118,551 all from Tocris (Bristol, UK). Epinephrine (INFECTOPHARM GmbH, Heppenheim, Germany).

HEPES solution: 20 mM HEPES, 4.5 mM KCl, 2.5 mM CaCl_2_, 11 mM Glucose, 140 mM NaCl, 1 mM MgCl_2_, pH was adjusted to 7.4 at 30°C or 4–8°C for tissue preparation using NaOH (4 M).

### Statistical analysis

Shapiro-Wilk test was used to test for normal distribution. Mann Whitney U-test was used to compare equivalent measuring points from different experiments.

## Results

### Adrenergic receptor mRNA expression in tracheal epithelium

RT-PCR experiments were used to identify the exact distribution of adrenergic receptor mRNA in whole tracheal walls and in isolated tracheal epithelium. Histological staining was used to verify the integrity of underlying cell structures after epithelium removal and purity of isolated epithelial cells.

**Fig 1:**
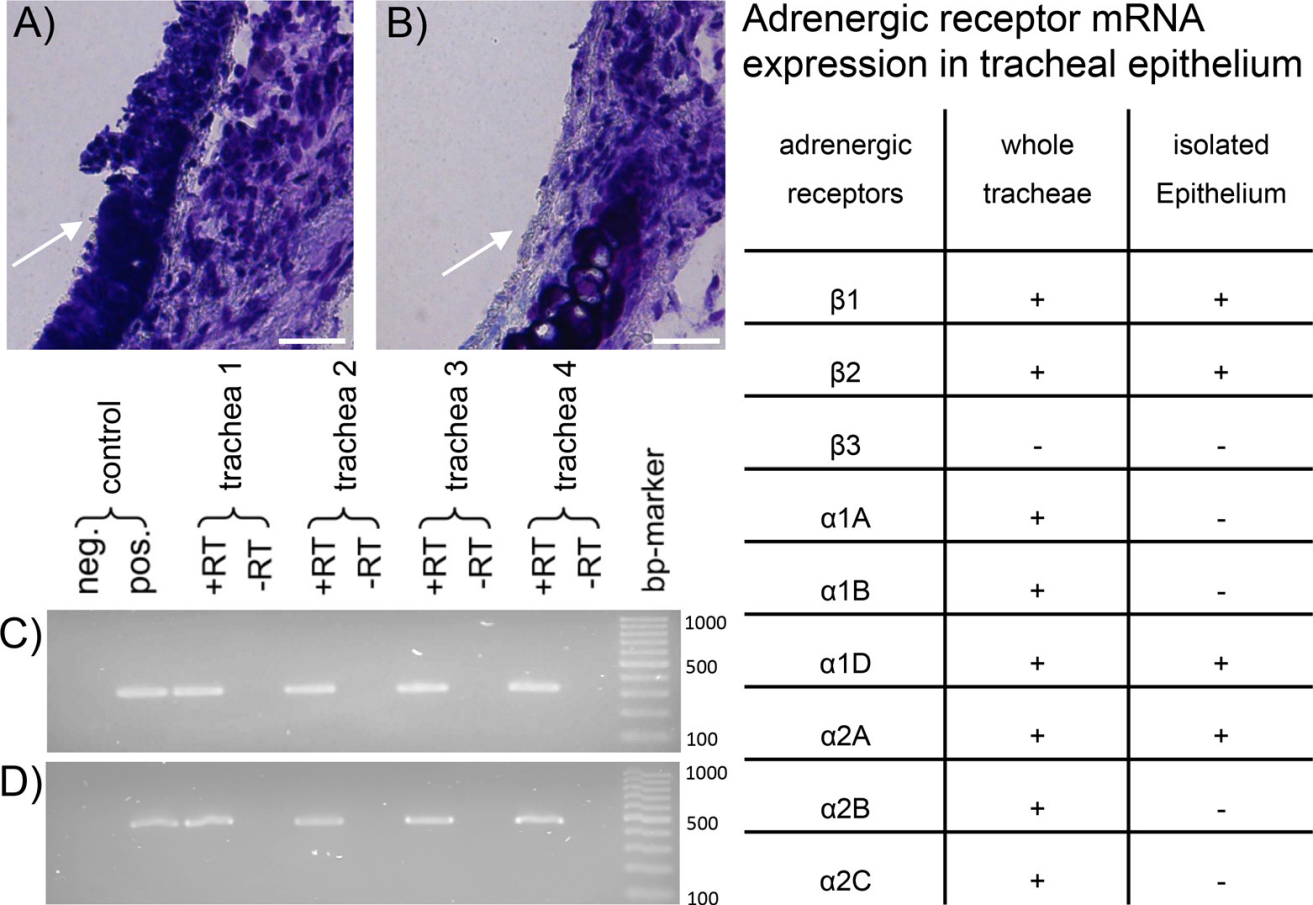
Adrenergic receptor mRNA expression in tracheal epithelium. A: Transversal section of untreated murine trachea displays complete and intact respiratory epithelium (arrow) as used for PTV experiments and for mRNA analysis from entire organs. B: Tracheal wall section after isolation of epithelium by brush removal shows the specificity of epithelial cell isolation for mRNA extraction. Thus, the arrow indicates a section of the trachea where the epithelium remained. All other tracheal wall structures were left intact. C & D: In isolated epithelium harvested from four different tracheae (1–4) mRNA of the adrenergic receptors β1(C), β2 (D) were found (α1D and α2A are not shown). epithelium: → scale bars: 25 μm. Table: Distribution of adrenergic receptors in the whole tracheae and in the isolated epithelium. +: mRNA expressed; -: mRNA not detectable.

Tracheae freshly prepared showed intact wall structures including the epithelium (Fig 1A, arrow). For RNA extraction, epithelial cells were isolated by gently brushing the longitudinally opened tracheae. This method was specific for epithelial cells since all remaining wall structures were left intact including the basal membrane (Fig 1B, arrow). In the tracheal epithelium we found mRNA of the adrenergic receptors β1, β2 (Fig 1C and 1D) and α1D, α2A. In contrast mRNA of further adrenergic receptors excluding the β3 receptor was detected in whole tracheal sections (see table in Fig 1).

### Epinephrine increases PTV through beta-adrenergic receptors

**Fig 2:**
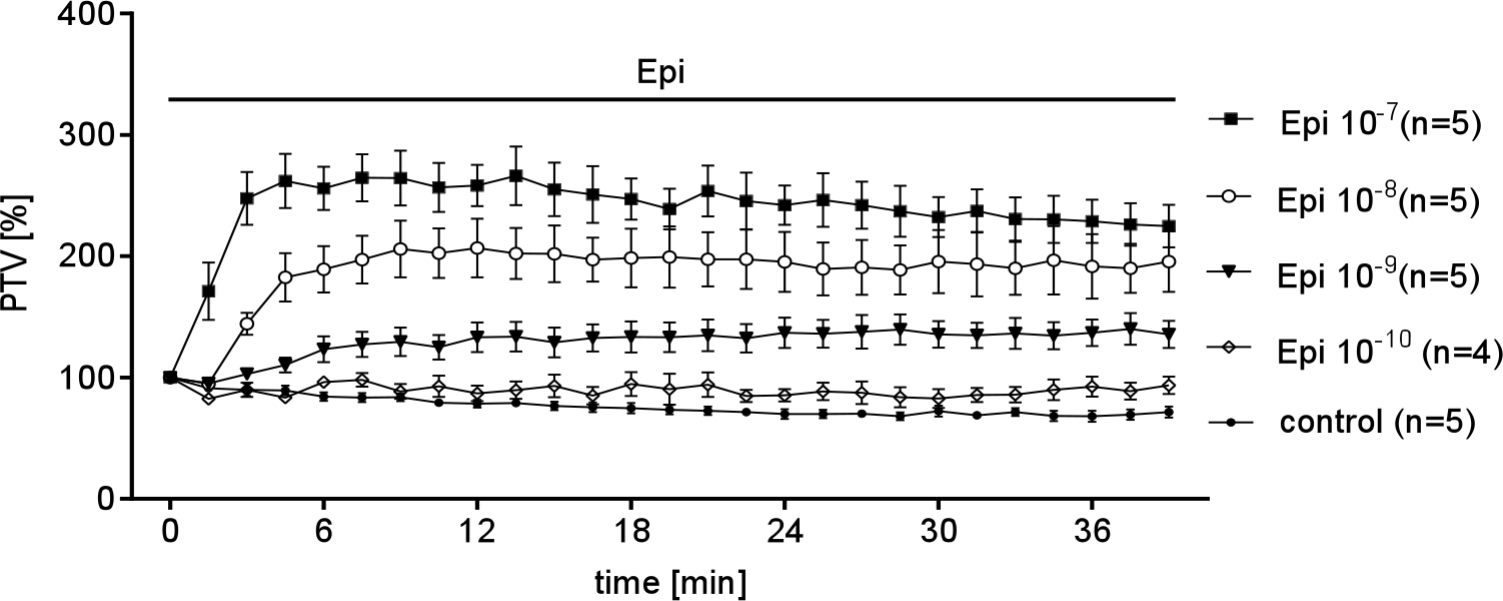
Concentration dependent Epi effects on PTV. Basic values of PTV were normalized to 100% at the initiation of Epi application at time point zero. Epi increases the PTV, which is already detectable 1.5 minutes later when using the highest concentrations (10^−7^ M). In contrast lower concentrations applied led to delayed acceleration of PTV that was first detectable after 3 min (10^−8^ M) or 4.5 min (10^−9^ M). All used concentrations evoked after an acceleration period to a steady state increase of the PTV. During prolonged application only little deceleration was registered this led to an approach of similar PTV when using high dosages. Further, the peak PTV was dose dependent (control: untreated tracheae (n = 5); Epi (10^−7^ M, n = 5); Epi (10^−8^ M, n = 5); Epi (10^−9^ M, n = 5); Epi (10^−10^ M, n = 4).

**Fig 3:**
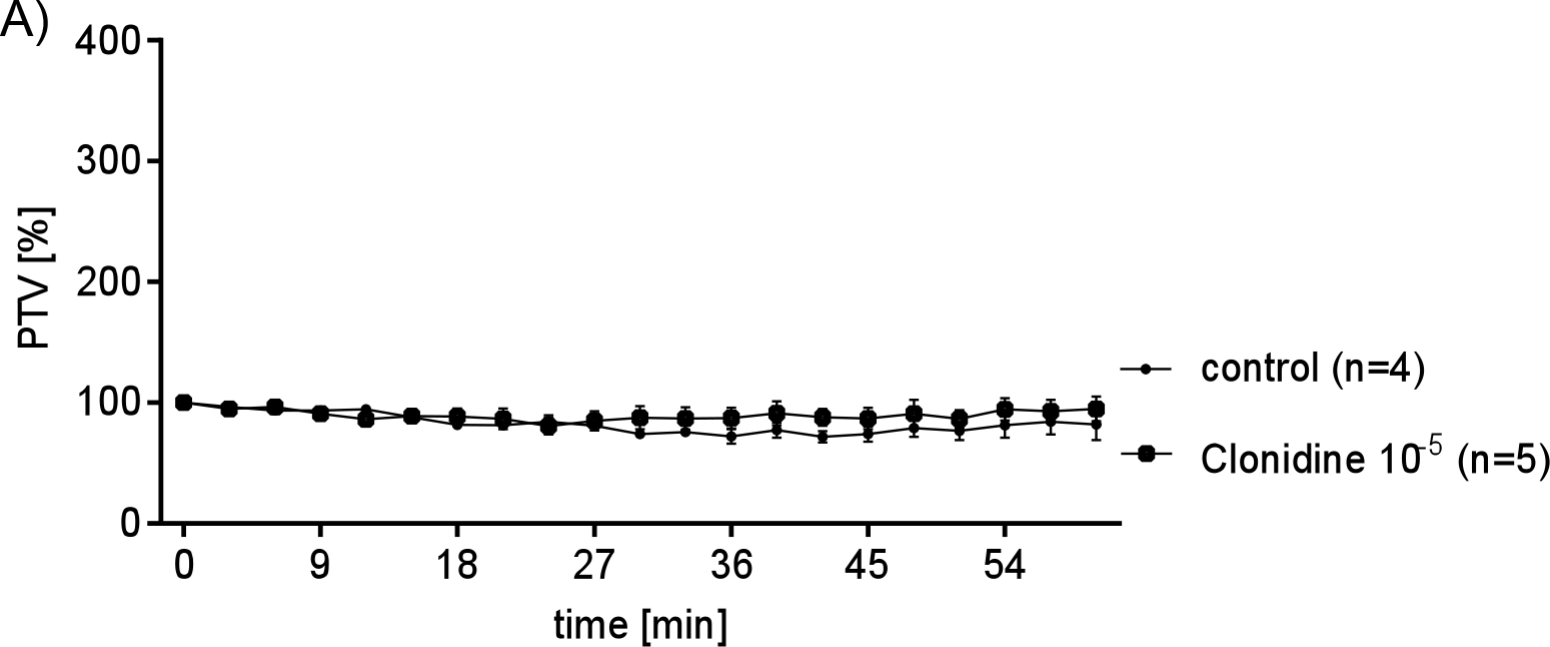
PTV increase is independent from α-receptor activation. Clonidine an activator of α-receptors was applied in a maximum concentration. However there was no effect onto PTV as a response to continuous clonidine concentration (10^−5^ M) when compared to control (untreated tracheae). Exposure period was set to 60 min (control: untreated tracheae (n = 4); clonidine (10^−5^ M, n = 5); n: tracheae from n animals).

**Fig 4:**
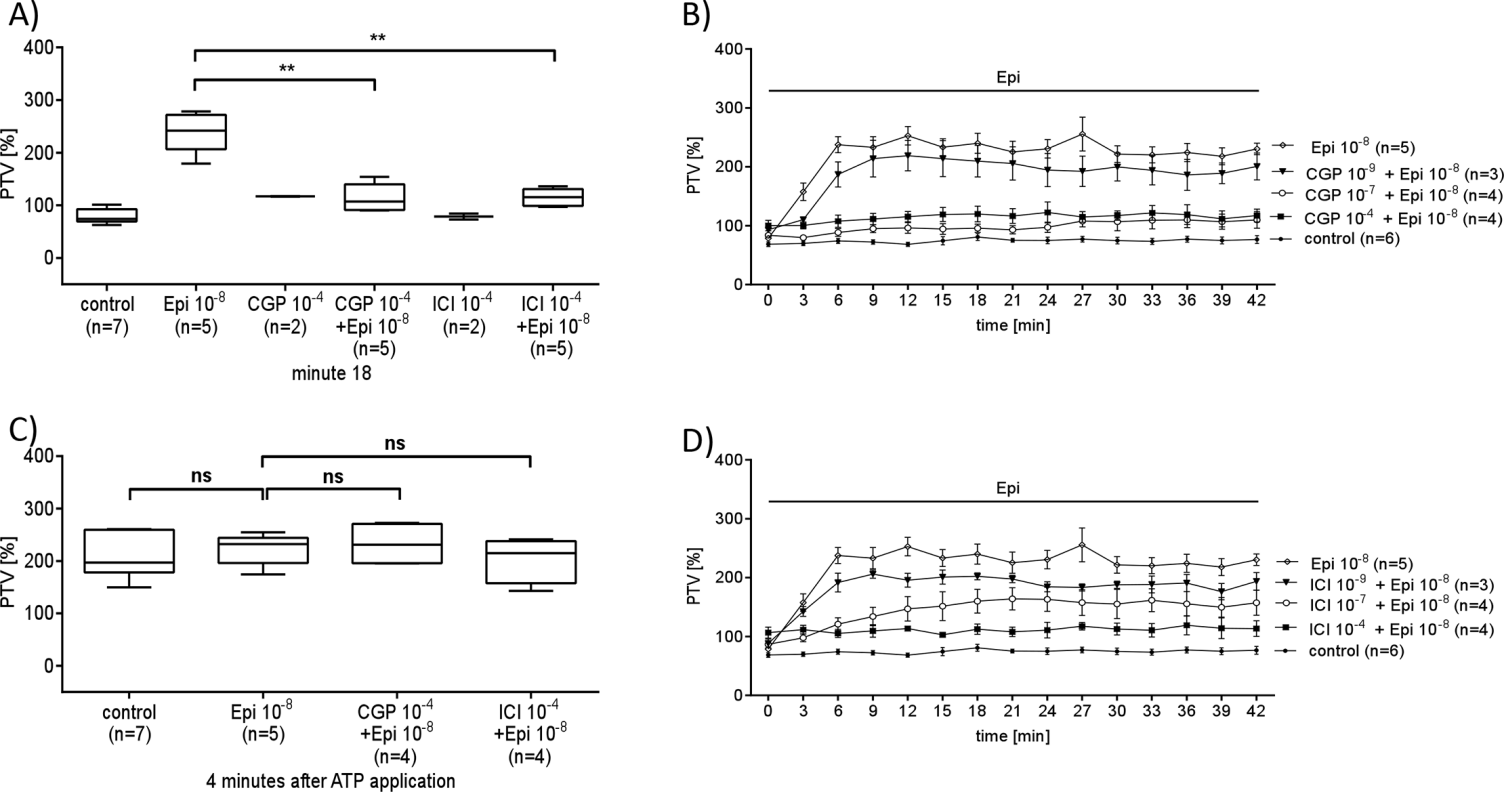
Pharmacological inhibition of β-receptors prevents the Epi induced increase in PTV. Pharmacological inhibition of either β1- or β2-receptors was used to inhibit the PTV response evoked by Epi (A) The β1-selective inhibitor CGP 20712 was applied in increasing concentrations. Low concentrations equal to Epi (10^−8^ M) only partially reduced the Epi-activated PTV. (B) Exposure to high concentration of CGP (10^−4^ M), when assumed that it binds non-specific, almost completely prevented the effect evoked by Epi (10^−8^ M, **p<0.01). (B) Exposure of tracheal epithelium to CGP 20712 has no effect when applied solely. However, high concentrations of CGP 20712 completely inhibited the response to Epi (10^−8^ M, **p<0.01). (C) The β2-selective inhibitor ICI 118.551 (10^−4^ M,) too has no agonistic effect when applied on its own, thus it completely prevented the increase in PTV evoked by Epi (10^−8^ M, **p<0.01) (Data compared 18 minutes after Epi application). Mann Whitney U-test. Box blots depict Mean ± S.E.M. (control: untreated tracheae (n = 7); Epi (10^−8^ M, n = 5); CGP (10^−4^ M, n = 2), CGP 10^−4^ M + Epi 10^−8^ M (n = 5); ICI (10^−4^ M, n = 2); ICI 10^−4^ M + Epi 10^−8^ M (n = 5), n: number of tracheae).

To determine whether alpha- or beta-adrenergic receptors regulate PTV, we added epinephrine or the alpha-agonist clonidine. Epinephrine (Epi10^-10^ to 10^−7^ M) caused a dose dependent increase in PTV (Fig 2). In contrast, the α-receptor agonist clonidine did not alter PTV (Fig 3). Because clonidine failed to stimulate PTV, we hypothesized that Epi controls PTV through beta-receptors. Pharmacological inhibition of β-receptors prevented the Epi (10^−8^ M) induced increase in PTV (Fig 4), showing that beta-receptor signaling is required.

### GM or Zym inhibit the Epi induced PTV increase

**Fig 5:**
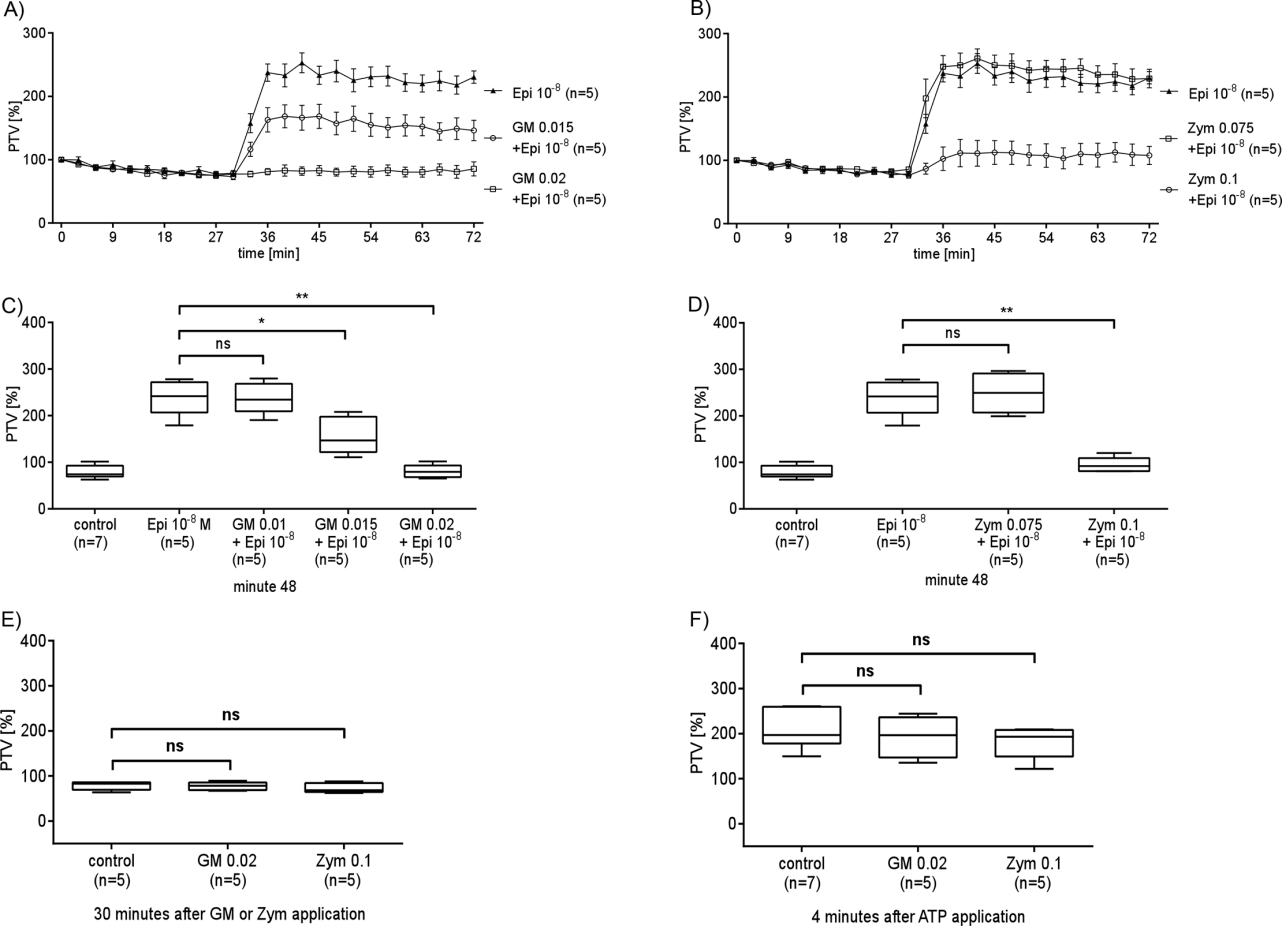
GM or Zym reduce the Epi induced PTV increase. 30 minute pre-incubation with GM (A) or Zym (B) reduced in a dose dependent manner the response to Epi 10^−8^ M. The inhibiting effect of GM (C) (0.015 mg/ml, 0.02 mg/ml) or Zym (D) (0.1 mg/ml) onto the response to Epi was significant when compared to experiments when only Epi was applied (*p<0.05, **p<0.01). Very low concentrations of GM 0.01 mg/ml (p = 0.9444) or Zym 0.075 mg/ml (p = 0.6667) did not alter the Epi evoked increase in PTV. (E) (F) Neither high concentrations of GM (0.02 mg/ml) nor of Zym (0.1 mg/ml) affected the PTV nor the response of ATP as an index of viability of individually investigated tracheae. Exposure periods are presented by horizontal bars. Mann Whitney U-test. Mean ± S.E.M.. (control: untreated tracheae (n = 7); all other experiments (n = 5), n: tracheae from n animals).

The fungal cell wall components GM (0.02 mg/mL) and Zym (0.1 mg/mL) had no effect on basal PTV (S1A and S1B Fig). However, 30 min pre-incubation with GM or Zym led to a dose dependent inhibition of the Epi (10^−8^ M) induced PTV increase (Fig 5A & 5B). GM concentrations of 0.015 mg/ml or 0.02 mg/ml significantly reduced the Epi induced PTV elevation (Fig 5C). Zym applied in a concentration of 0.1 mg/ml almost completely decreased the Epi induced PTV (Fig 5D). Lower concentrations of GM or Zym reduced the Epi effect only partially or had no significant impact on the Epi induced PTV.

### Interference of GM or Zym with the Epi cascade

**Fig 6:**
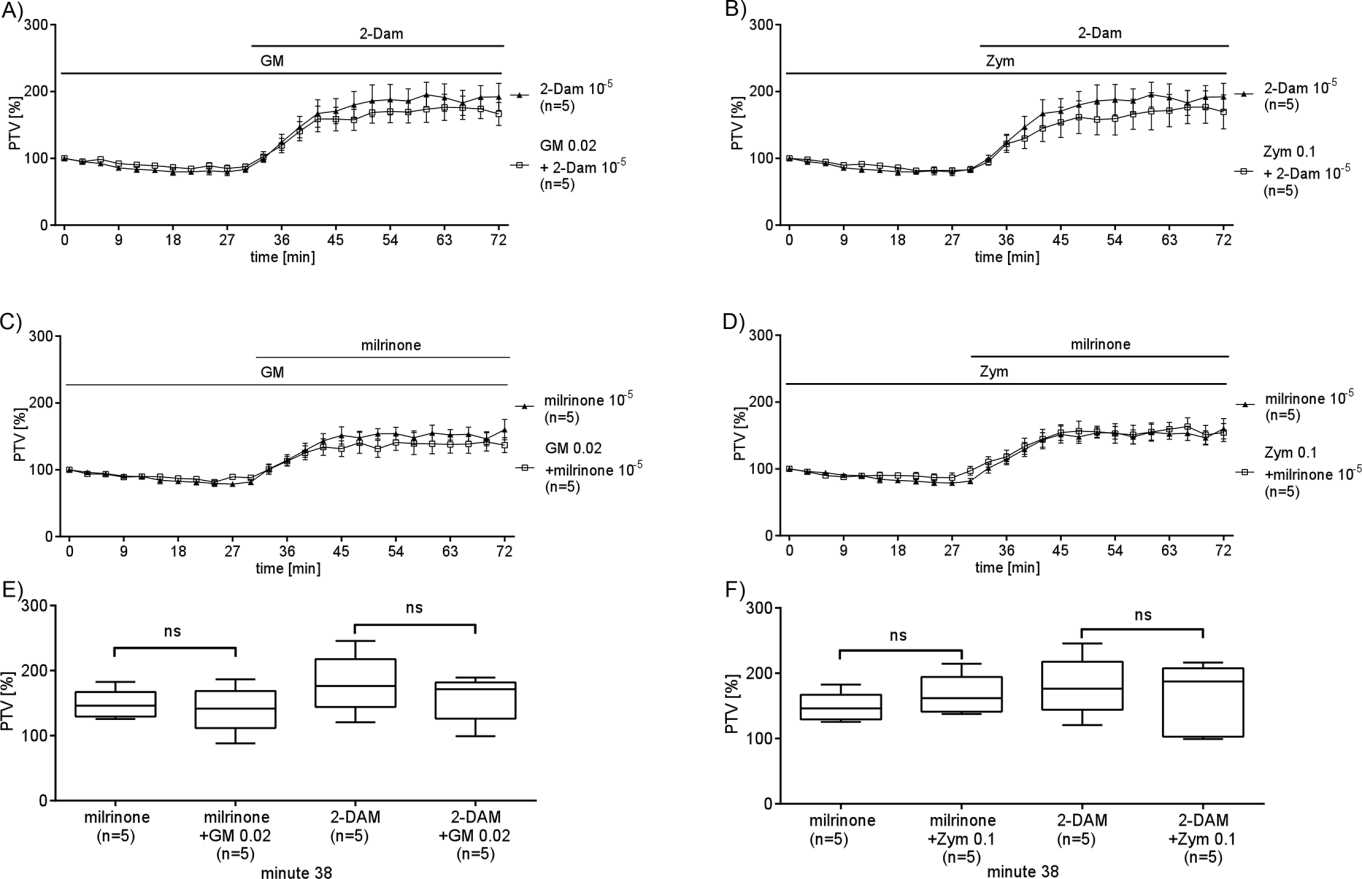
GM or Zym do not influence the adenylyl cyclase pathway. Direct activation of intracellular adenylyl cyclase using 2-DAM (10^−5^ M) significantly increased PTV. This accelerated PTV was neither reduced by pre-incubation with high concentrations of GM 0.02 mg/ml (A) nor with Zym 0.1 mg/ml (B). (C & D) As well the reduced cAMP degradation by inhibition of phosphodiesterase 3 using milrinone (10^−5^ M) significantly increased the PTV. Too this increase could neither be affected by GM (0.02 mg/ml) nor by Zym (0.1 mg/ml). E & F show the boxplots of PTV values after PTV had reached a stable plateau. There was no difference in PTV under application of AC inhibitors or PDE inhibitor alone or in combination with GM or Zym. Mean ± S.E.M. Milrinone 10^−5^ M vs. milrinone 10^−5^ M + GM 0.02 mg/ml (p = 0.9444); 2-DAM 10^−5^ M vs 2-DAM 10^−5^ M + GM 0.02 mg/ml (p = 0.4524); milrinone 10^−5^ M vs. milrinone 10^−5^ M + Zym 0.1 mg/ml (p = 0.4127); 2-DAM 10^−5^ M vs 2-DAM 10^−5^ M + Zym 0.1 mg/ml (p = 0.8016). Mann Whitney U-test. (n = 5 for all experiments, n: tracheae from n animals).

To determine whether Zym or GM interfere with adrenergic signaling upstream or downstream of receptor binding we stimulated intracellular components of the Epi signaling cascade. The activator of the adenylyl cyclase 2-DAM (10^−5^ M) or milrinone (10^−5^ M, an inhibitor of the phosphodiesterase 3, were applied to enhance PTV in order to elucidate the location of Zym or GM interference. GM or Zym were applied 30 minutes before application of milrinone or 2-DAM (See Fig 6A–6D). GM (0.02 mg/ml, Fig 6A and 6C) neither reduced the response to milrinone nor the response to 2-DAM. Further treatment with Zym (0.1 mg/ml, Fig 6B and 6D) had no effect onto the response to milrinone or 2-Dam. Thus GM or Zym did not interfere within the epinephrine cascade downstream of the adenylyl cyclase.

### GM or Zym induce ROS production

**Fig 7:**
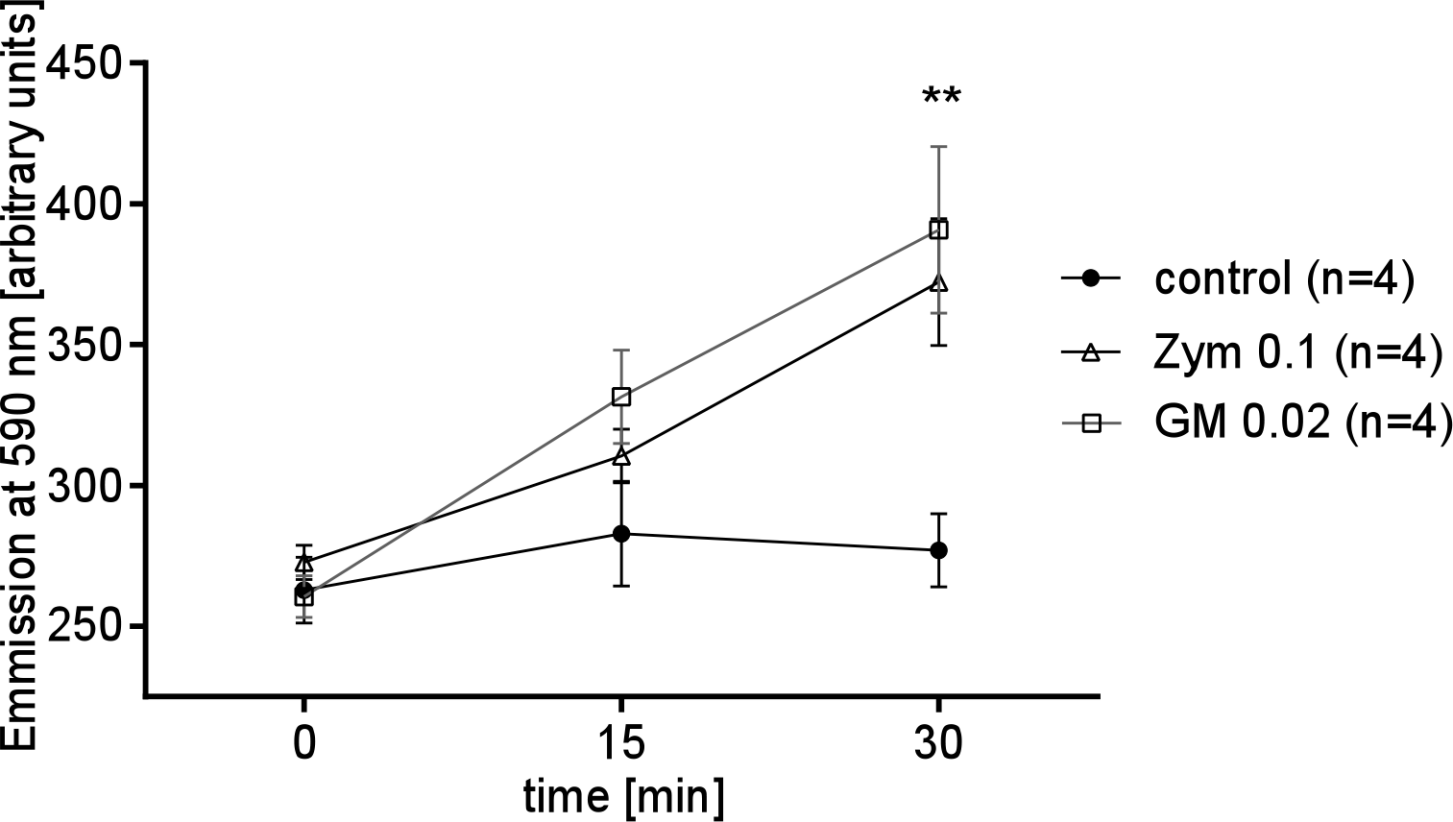
GM or Zym induce ROS generation. In tracheae incubated with GM or Zym ROS generation was detected that further increased under prolonged exposure periods and became highly significant after 30 minutes when compared to control organs (**p<0.01). Mann Whitney U-test. Mean ± S.E.M.. (In all experiments n = 4, n: tracheae from n animals).

We hypothesized that GM and Zym elicit ROS production, potentially inhibiting beta-adrenergic receptor signaling by either degrading epinephrine or modifying its receptor. Amplex Red analysis showed that both GM and Zym addition significantly increased ROS generation compared to untreated control trachea (Fig 7).

### ROS scavenging by N-acetylcysteine and Tempol reduce the effect of GM or Zym

**Fig 8:**
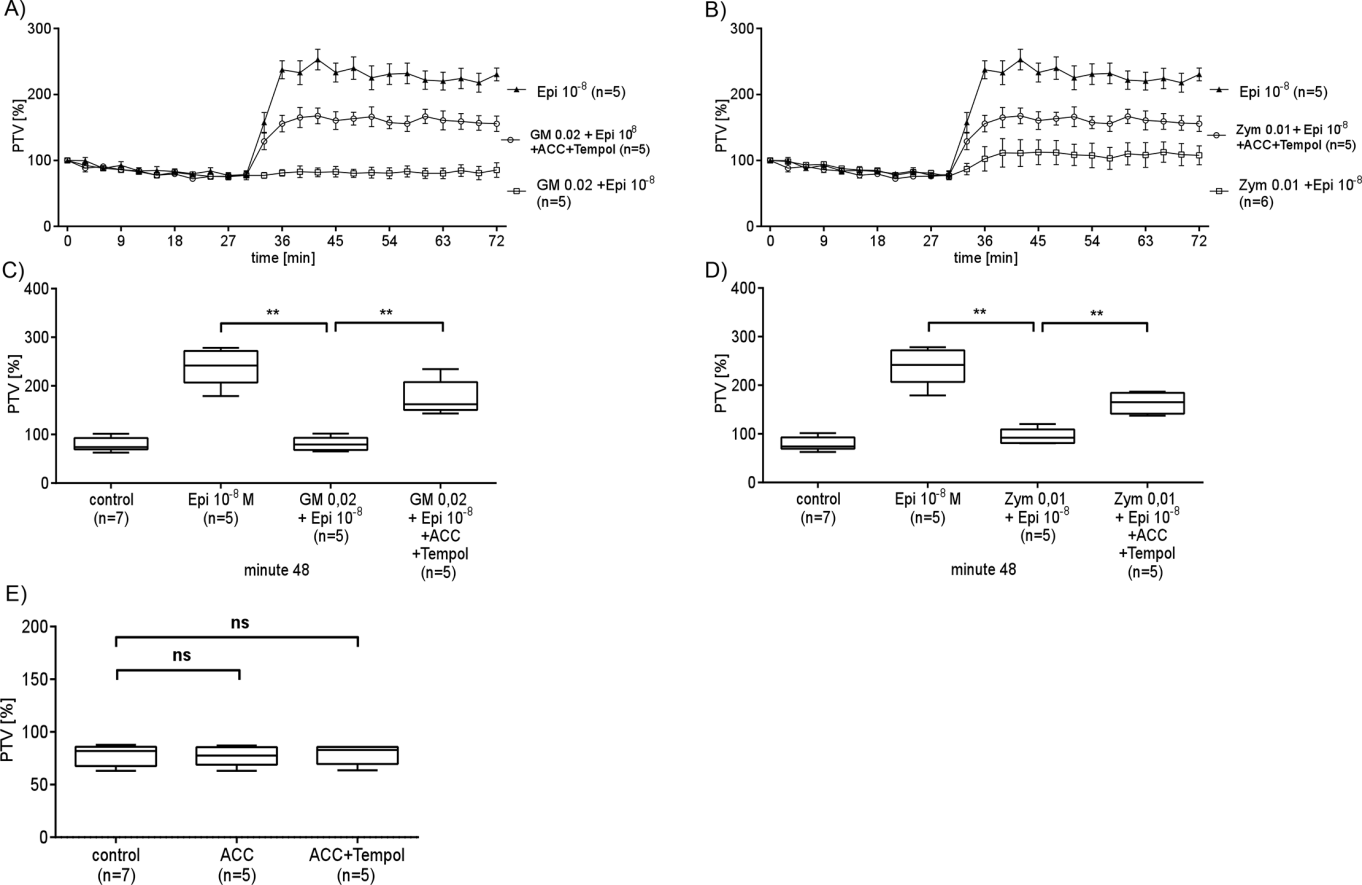
ROS scavenging reduces the effect of GM or Zym. Simultaneous Application of the ROS scavengers ACC (20 mM) and Tempol (5 mM) in the presence of GM (0.02 mg/ml) restored the Epi (10^−8^ M) induced PTV. This increase in PTV was significantly higher in comparison to tracheae which were not pretreated with ROS scavengers (**p<0.01; A & C). (B & D) The inhibiting effect by Zym (0.1 mg/ml) onto Epi (10^−8^ M) evoked PTV-increase was also reduced by the simultaneous application of the ROS scavengers ACC and Tempol (**p<0.01; B & D). (E) Application of ROS scavengers ACC (20 mM) alone or in combination with Tempol (5 mM) did not change PTV. Mann Whitney U-test. Mean ± S.E.M.. (control: n = 7; all other experiments n = 5, n: tracheae from n animals).

The ROS scavengers N-acetylcysteine (ACC, 20 mM) and Tempol (5 mM) restored epinephrine responsiveness of PTV in GM- (0.02 mg/ml) and Zym (0.1 mg/ml) -treated tracheas (Fig 8A & 8B). Tracheae treated with Epi and GM or Zym showed a significant increase in PTV after previous simultaneous application of ACC and Tempol compared to tracheae not treated with ROS scavengers (Fig 8C & 8D).

## Discussion

Here we investigated the adrenergic signal cascade that activates cilia transport capacity in murine tracheal epithelium and its modulation by fungal cell wall components.

Therefore we first analyzed the distribution of adrenergic receptor mRNA in entire tracheal walls in comparison to isolated tracheal epithelium, demonstrated in Fig 1. The RT-PCR experiments clearly provided evidence that mRNA of all investigated adrenergic receptors except β3, are expressed in tracheal walls. To identify receptors that are expressed in the epithelium, we carefully isolated the epithelial cell layer before mRNA extraction. Histological sections confirmed the specificity of this epithelial cell layer isolation. The subsequent mRNA extraction followed by RT-PCR analysis revealed that only mRNAs of β1, β2, α1D and α2A adrenergic receptors are expressed in the tracheal epithelium.

We further used pharmacological agents to verify the identified receptors by their influence onto cilia function via measuring the dose dependent changes in PTV as a response to Epi (Fig 2) or clonidine (Fig 3). Indeed, Epi evoked a dose dependent increase of the PTV, while the selective α-stimulator clonidine had no effect. The latter results probably exclude a substantial contribution of α receptors to cilia function, whereas β1 and β2 receptors seem to be responsible for the observed effect. To further prove this assumption selective pharmacological inhibitors of β1 or β2 receptors were applied. With this experimental setup we confirmed that inhibition of β1 using CGP 20712 as well as inhibition of β2 using ICI 118.551 indeed inhibited the Epi induced increase in PTV. Consequently Epi response is transmitted either by β1 or β2 receptors expressed in tracheal epithelium.

Previous studies already demonstrated that different physiological modulators can directly modify the PTV. Activation of muscarinic acetylcholine receptor M3 upregulates cilia-driven particle transport, while stimulation of the M2 receptor decreases ciliary transport [9]. Apart from these modulators further signal molecules are known to indirectly change the activity of the mucociliary apparatus [19]. For example tumor necrosis factor alpha an inflammatory response molecule elicits the effect of serotonin at tracheal ciliated cells [13]. Beyond that, the lower airways are in constant contact to pathogens that may also influence the function and integrity of the ciliary clearing mechanism. Here we investigated the effect of GM and Zym. Both molecules act as exoantigens and can be found in infected organisms as well as in lower airways [20].

We tried to elucidate their potential pathogenic role in lower airways by investigating their effect onto PTV. Under basal conditions both did not alter PTV. However, when PTV was stimulated by Epi to physiological rates or beyond, GM as well as Zym reduced the effect of Epi onto PTV in a dose dependent manner.

We used pharmacologic tools to detect possible locations within the catecholamine cascade, including the adenylyl cyclase pathway. Using 2-DAM, a direct activator of the adenylyl cyclase, resulted in increased PTV. However, neither GM nor Zym impaired the response to 2-DAM. Stepping further downstream in the catecholamine cascade we also performed experiments with milrinone. This inhibitor of phosphodiesterase 3 causes enhanced cAMP concentrations [21], [22] and here it was sufficient to maximally stimulate PTV. High concentrations of cAMP lead also to an activation of an axonemal protein kinase A which directly phosphorylates dynein chains that are components of the microtubules acting as an ATPase propelling the cilia motion as a part of the axoneme [23]. In the present study neither application of GM nor of Zym were able to reduce or inhibit the milrinone augmented PTV. Overall neither a direct stimulation of the adenylyl cyclase nor an indirect increase in cAMP were influenced by GM or Zym suggesting that both molecules affect the Epi signal cascade upstream of the adenylyl cyclase (Fig 6).

It is known that GM and Zym induce ROS generation by activation of NADPH oxidases in different organs as well as in lungs resulting in altered cell functions or signal cascades [24], [25]. When GM or Zym were applied onto tracheae we detected high levels of ROS concentrations generated as a function of time (Fig 7). Under our basal conditions, when PTV is lower than in vivo, both GM and Zym do not affect particle transport even at high concentrations, suggesting ROS do not alter these low activation states. In different cell types ROS enhance the activity of β2-receptors or G-protein depending signal cascades; however this may not be a universal model. Since we observed in the present study that the Epi induced increase of PTV via activation of β1 or β2 receptors was reduced by GM or Zym.

Most likely Epi is directly oxidized by ROS because catecholamines act like ROS scavengers even more efficiently like classical scavengers which then results in a reduced activity or binding capacity to adrenergic receptors as previously described [26] [27]. Eventually the physiological Epi induced activation of PTV is inhibited by endogenous ROS generated under the influence of GM or Zym that oxidize and inactivate Epi in a concentration dependent manner. This assumption is strengthened by experiments in which we added different ROS scavengers that were able to almost completely restore the Epi effect in the presence of GM or Zym (Fig 8).

Despite the mounting evidence about the potential underlying mechanism, we are well aware of the limitations of our study. In fact, the final experimental proof of the “endogenous counter-catecholaminergic ROS pathway” is still lacking in our results and needs to be subject to further research, potentially using approaches like transgenic animals or non-oxidizable derivatives of epinephrine we do not have access to. Despite the last missing link we are sure that the mechanism we unraveled so far has a tremendous translational potential in the context of intensive care medicine or pneumology.

In summary, our findings show the direct impact of the fungal cell wall components, GM and Zym, to the functional ciliary transport mechanism. As Fungal airway colonization of *Candida albicans* is known to provoke the development of bacterial pneumonia in rats [28], a better understanding of fungal effects onto the lower respiratory tract might help to develop new antifungal therapeutic concepts.

Our study clearly revealed the mechanism of adrenergic cilia activation in murine trachea via β1 or β2 receptors expressed in tracheal epithelium. This signal transduction cascade is inhibited by the fungal exoantigens GM or Zym triggering ROS generation that probably leads to an oxidization of Epi which then mounts in a reduced binding affinity, a chain of action which can be counteracted by pre-incubation with ROS scavengers.

## Supporting Information

S1 Fig A & B: GM or Zym had no effect on basal PTV. (TIF)

S1 File Raw data of Fig 2. (XLSX)

S2 File Raw data of Fig 3. (XLSX)

S3 File Raw data of Fig 4. (XLSX)

S4 File Raw data of Fig 5. (XLSX)

S5 File Raw data of Fig 6. (XLSX)

S6 File Raw data of Fig 7. (XLSX)

S7 File Raw data of Fig 8. (XLSX)

S8 File Raw data of S1A and S1B Fig. (XLSX)

S1 Table List of primer used for PCR. (DOCX)

## References

[1] Knowles MR, Boucher RC (2002) Mucus clearance as a primary innate defense mechanism for mammalian airways. J Clin Invest 109: 571–577. 11877463 10.1172/JCI15217 PMC150901

[2] Lee TW, Southern KW (2012) Topical cystic fibrosis transmembrane conductance regulator gene replacement for cystic fibrosis-related lung disease. Cochrane Database Syst Rev 10: CD005599 10.1002/14651858.CD005599.pub3 23076917

[3] Pezzulo AA, Tang XX, Hoegger MJ, Alaiwa MH, Ramachandran S, Moninger TO, et al (2012) Reduced airway surface pH impairs bacterial killing in the porcine cystic fibrosis lung. Nature 487: 109–113. 10.1038/nature11130 22763554 PMC3390761

[4] Hoegger MJ, Fischer AJ, McMenimen JD, Ostedgaard LS, Tucker AJ, Awadalla MA, et al (2014) Impaired mucus detachment disrupts mucociliary transport in a piglet model of cystic fibrosis. Science 345: 818–822. 10.1126/science.1255825 25124441 PMC4346163

[5] Stoltz DA, Meyerholz DK, Welsh MJ (2015) Origins of cystic fibrosis lung disease. N Engl J Med 372: 1574–1575. 10.1056/NEJMc1502191#SA1 25875271

[6] Keiser NW, Birket SE, Evans IA, Tyler SR, Crooke AK, Sun X, et al (2015) Defective innate immunity and hyperinflammation in newborn cystic fibrosis transmembrane conductance regulator-knockout ferret lungs. Am J Respir Cell Mol Biol 52: 683–694. 10.1165/rcmb.2014-0250OC 25317669 PMC4491130

[7] Birket SE, Chu KK, Liu L, Houser GH, Diephuis BJ, Wilsterman EJ, et al (2014) A functional anatomic defect of the cystic fibrosis airway. Am J Respir Crit Care Med 190: 421–432. 10.1164/rccm.201404-0670OC 25029666 PMC4214131

[8] Salathe M (2007) Regulation of mammalian ciliary beating. Annu Rev Physiol 69: 401–422. 16945069 10.1146/annurev.physiol.69.040705.141253

[9] Klein MK, Haberberger RV, Hartmann P, Faulhammer P, Lips KS, Krain B, et al (2009) Muscarinic receptor subtypes in cilia-driven transport and airway epithelial development. Eur Respir J 33: 1113–1121. 10.1183/09031936.00015108 19213795 PMC3895332

[10] Barnes PJ, Basbaum CB (1983) Mapping of adrenergic receptors in the trachea by autoradiography. Exp Lung Res 5: 183–192. 6317370 10.3109/01902148309061513

[11] Abraham G, Kottke C, Dhein S, Ungemach FR (2003) Pharmacological and biochemical characterization of the beta-adrenergic signal transduction pathway in different segments of the respiratory tract. Biochem Pharmacol 66: 1067–1081. 12963495 10.1016/s0006-2952(03)00460-x

[12] Verdugo P, Johnson NT, Tam PY (1980) beta-Adrenergic stimulation of respiratory ciliary activity. J Appl Physiol Respir Environ Exerc Physiol 48: 868–871. 7451296 10.1152/jappl.1980.48.5.868

[13] Weiterer S, Schulte D, Muller S, Kohlen T, Uhle F, Weigand MA, et al (2014) Tumor necrosis factor alpha induces a serotonin dependent early increase in ciliary beat frequency and epithelial transport velocity in murine tracheae. PLoS One 9: e91705 10.1371/journal.pone.0091705 24626175 PMC3953516

[14] Delhaes L, Monchy S, Frealle E, Hubans C, Salleron J, Leroy S, et al (2012) The airway microbiota in cystic fibrosis: a complex fungal and bacterial community—implications for therapeutic management. PLoS One 7: e36313 10.1371/journal.pone.0036313 22558432 PMC3338676

[15] Mear JB, Gosset P, Kipnis E, Faure E, Dessein R, Jawhara S, et al (2014) Candida albicans airway exposure primes the lung innate immune response against Pseudomonas aeruginosa infection through innate lymphoid cell recruitment and interleukin-22-associated mucosal response. Infect Immun 82: 306–315. 10.1128/IAI.01085-13 24166952 PMC3911865

[16] Becker MJ, Lugtenburg EJ, Cornelissen JJ, Van Der Schee C, Hoogsteden HC, De Marie S, et al (2003) Galactomannan detection in computerized tomography-based broncho-alveolar lavage fluid and serum in haematological patients at risk for invasive pulmonary aspergillosis. Br J Haematol 121: 448–457. 12716367 10.1046/j.1365-2141.2003.04308.x

[17] Dostert C, Tschopp J (2007) DEteCTINg fungal pathogens. Nat Immunol 8: 17–18. 17179965 10.1038/ni0107-17

[18] Whitmore LC, Goss KL, Newell EA, Hilkin BM, Hook JS, Moreland JG, et al (2014) NOX2 protects against progressive lung injury and multiple organ dysfunction syndrome. Am J Physiol Lung Cell Mol Physiol 307: L71–82. 10.1152/ajplung.00054.2014 24793165 PMC4080282

[19] Konig P, Krain B, Krasteva G, Kummer W (2009) Serotonin increases cilia-driven particle transport via an acetylcholine-independent pathway in the mouse trachea. PLoS One 4: e4938 10.1371/journal.pone.0004938 19290057 PMC2654158

[20] Affolter K, Tamm M, Jahn K, Halter J, Passweg J, Hirsch HH, et al (2014) Galactomannan in bronchoalveolar lavage for diagnosing invasive fungal disease. Am J Respir Crit Care Med 190: 309–317. 10.1164/rccm.201403-0431OC 25007380

[21] Alousi AA, Johnson DC (1986) Pharmacology of the bipyridines: amrinone and milrinone. Circulation 73: III10-24. 2417744

[22] Smith SN, Middleton PG, Chadwick S, Jaffe A, Bush KA, Rolleston S, et al (1999) The in vivo effects of milrinone on the airways of cystic fibrosis mice and human subjects. Am J Respir Cell Mol Biol 20: 129–134. 9870926 10.1165/ajrcmb.20.1.3278

[23] Wyatt TA, Spurzem JR, May K, Sisson JH (1998) Regulation of ciliary beat frequency by both PKA and PKG in bovine airway epithelial cells. Am J Physiol 275: L827–835. 9755116 10.1152/ajplung.1998.275.4.L827

[24] Makni-Maalej K, Chiandotto M, Hurtado-Nedelec M, Bedouhene S, Gougerot-Pocidalo MA, Dang PM, et al (2013) Zymosan induces NADPH oxidase activation in human neutrophils by inducing the phosphorylation of p47phox and the activation of Rac2: involvement of protein tyrosine kinases, PI3Kinase, PKC, ERK1/2 and p38MAPkinase. Biochem Pharmacol 85: 92–100. 10.1016/j.bcp.2012.10.010 23085266

[25] Rementeria A, Lopez-Molina N, Ludwig A, Vivanco AB, Bikandi J, Pontòn J, et al (2005) Genes and molecules involved in Aspergillus fumigatus virulence. Rev Iberoam Micol 22: 1–23. 15813678 10.1016/s1130-1406(05)70001-2

[26] Wang L, Fu X, Zeng Y, Zhu S (2014) Epinephrine promotes development potential of vitrified mouse oocytes. Pak J Biol Sci 17: 254–259. 24783810 10.3923/pjbs.2014.254.259

[27] Shimizu T, Nakanishi Y, Nakahara M, Wada N, Moro-Oka Y, Hirano T, et al (2010) Structure Effect on Antioxidant Activity of Catecholamines toward Singlet Oxygen and Other Reactive Oxygen Species in vitro. J Clin Biochem Nutr 47: 181–190. 10.3164/jcbn.09-112 21103026 PMC2966927

[28] Roux D, Gaudry S, Khoy-Ear L, Aloulou M, Phillips-Houlbracq M, Bex J, et al (2013) Airway fungal colonization compromises the immune system allowing bacterial pneumonia to prevail. Crit Care Med 41: e191–199. 10.1097/CCM.0b013e31828a25d6 23887232

