# Supplementary figures and images for: Galactomannan and Zymosan Block the Epinephrine-Induced Particle Transport in Tracheal Epithelium
Source: PLoS One. 2015 Nov 16;10(11):e0143163. doi: 10.1371/journal.pone.0143163 (PMC4646458; doi:10.1371/journal.pone.0143163)

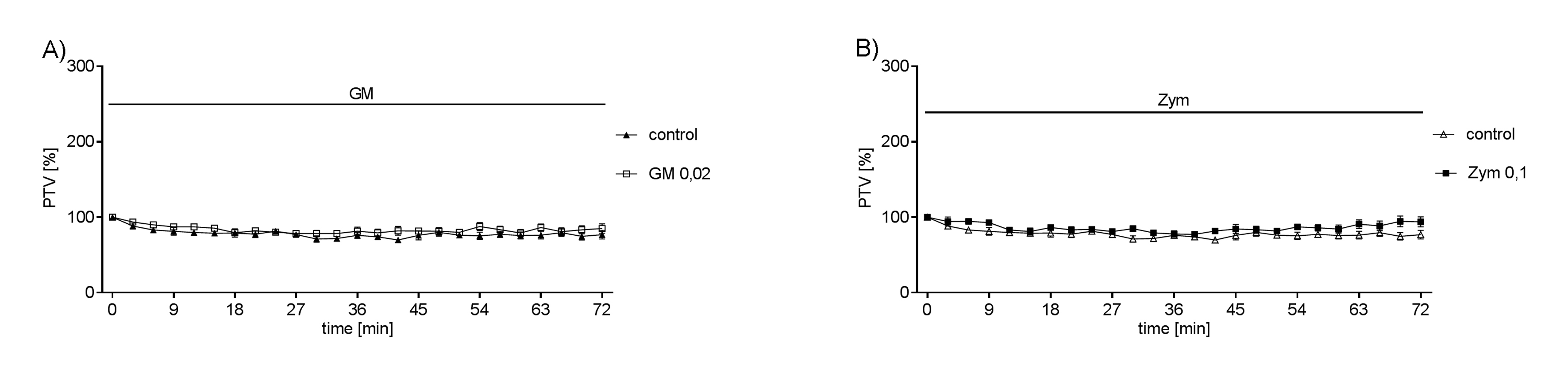

Supplement: S1 Fig — (TIF) [file pone.0143163.s001.tif]
