# Supplementary material for: Galactomannan and Zymosan Block the Epinephrine-Induced Particle Transport in Tracheal Epithelium
Source: PLoS One. 2015 Nov 16;10(11):e0143163. doi: 10.1371/journal.pone.0143163 (PMC4646458; doi:10.1371/journal.pone.0143163)
Supplement: S1 Table — (DOCX) [file pone.0143163.s010.docx]

S1 Table: List of primer used for PCR

| **Primer** | **Sequence** | **Base pare** | **Temperature** | **MgCl_2_** | **Cycles** |
| --- | --- | --- | --- | --- | --- |
| **ADRA1A** | s: CAG GAG AAG CAA GAA GCG A |  |  |  |  |
|  | as: CCG GCA GTG ACT CTC AAC TT | 311 | 60,5°C | 2mM | 34 |
| **ADRA1B** | s: CAC CCT CAG CAG TAC CAA GG |  |  |  |  |
|  | as: CTG TTG AAG TAG CCC AGC CA | 234 | 55,7°C | 2mM | 37 |
| **ADRA1D** | s: TGG TAT CTG TGG GAC CGC TA |  |  |  |  |
|  | as: GAA CCA GCA CAG GAC GAA GA | 410 | 60,5°C | 2mM | 37 |
| **ADRA2A** | s: AAG GCC ATC ATT GTC ACC GT |  |  |  |  |
|  | as: ACG CTT GGC GAT CTG GTA AA | 225 | 55,7°C | 2mM | 37 |
| **ADRA2B** | s: CAA GCC TCC AAG GGA GAA GG |  |  |  |  |
|  | as: CCA CGT TCG AGA AGG TCC AA | 851 | 60,5°C | 3mM | 37 |
| **ADRA2C** | s: CTG GTA CTT CGG GCA AGT GT |  |  |  |  |
|  | as: GTA GAA CGA GAC GAG AGG CG | 226 | 60,5°C | 2mM | 37 |
| **ADRB1** | s: CTG GTC ATG GGA TTG CTG GT |  |  |  |  |
|  | as: AGC ACT TGG GGT CGT TGT AG | 334 | 57,8°C | 2mM | 37 |
| **ADRB2** | s: GGT TAT CGT CCT GGC CAT CGT GTT TG |  |  |  |  |
|  | as: GTT CGT GAA GAA GTC ACA GCA AGT CTC | 468 | 59,1°C | 2mM | 37 |
| **ADRB3** | s: TCT AGT TCC CAG CGG AGT TTT CAT CG |  |  |  |  |
|  | as: CGC GCA CCT TCA TAG CCA TCA AAC C | 234 | 60,5°C | 1mM | 34 |
| **ACTB** | s: GTC CCT CAC CCT CCC AAA G |  |  |  |  |
|  | as: GCT GCC TCA ACA CCT CAA CCC | 266 | 60,5°C | 2mM | 37 |
